# Current Advances in Checkpoint Inhibitors: Lessons from Non-Central Nervous System Cancers and Potential for Glioblastoma

**DOI:** 10.3389/fonc.2017.00141

**Published:** 2017-07-06

**Authors:** Natasha Lakin, Robert Rulach, Stefan Nowicki, Kathreena M. Kurian

**Affiliations:** ^1^Brain Tumour Research Group, Institute of Clinical Neurosciences, Level 1, Learning and Research Building, Southmead Hospital, University of Bristol, Bristol, United Kingdom; ^2^The Beatson West of Scotland Cancer Centre, Glasgow, United Kingdom

**Keywords:** glioblastoma, immunotherapy, radiotherapy, ipilimumab, pembrolizumab, nivolumab

## Abstract

The adaptive immune system depends on the sequence of antigen presentation, activation, and then inhibition to mount a proportionate response to a threat. Tumors evade the immune response partly by suppressing T-cell activity using immune checkpoints. The use of cytotoxic T-lymphocyte-associated protein 4 (CTLA-4), programmed cell death 1 (PD-1), and programmed cell death ligand 1 (PD-L1) antibodies counteract this suppression, thereby enhancing the antitumor activity of the immune system. This approach has proven efficacy in melanoma, renal cancer, and lung cancer. There is growing evidence that the central nervous system is accessible to the immune system in the diseased state. Moreover, glioblastomas (GBMs) attract CTLA-4-expressing T-cells and express PD-L1, which inhibit activation and continuation of a cytotoxic T-cell response, respectively. This may contribute to the evasion of the host immune response by GBM. Trials are in progress to determine if checkpoint inhibitors will be of benefit in GBM. Radiotherapy could also be helpful in promoting inflammation, enhancing the immunogenicity of tumors, disrupting the blood–brain barrier and creating greater antigen release. The combination of radiotherapy and checkpoint inhibitors has been promising in preclinical trials but is yet to show efficacy in humans. In this review, we summarize the mechanism and current evidence for checkpoint inhibitors in gliomas and other solid tumors, examine the rationale of combining radiotherapy with checkpoint inhibitors, and discuss the potential benefits and pitfalls of this approach.

## Introduction

The recent exciting developments in immune checkpoint inhibitors have opened novel therapeutic avenues in previously intractable cancers. This has resulted in striking clinical benefits particularly in melanoma and renal-cell cancer (1–3). There are many checkpoint molecules that regulate the immune system to prevent over or under activation. Checkpoint inhibitors such as nivolumab or pembrolizumab (programmed cell death 1 or PD-1 inhibitors) and ipilimumab (a cytotoxic T-lymphocyte-associated protein 4 or CTLA-4 inhibitor) enhance the immune response to a tumor. Glioblastomas (GBMs) have multiple immunosuppressive effects involving programmed cell death ligand 1 (PD-L1) (4) and CTLA-4 checkpoints (5). The use of checkpoint inhibitors to upregulate the immune response in GBMs is under investigation in several clinical trials.

This paper aims to review the immune system in the central nervous system (CNS), how GBM evades it, and the evidence for checkpoint inhibition in non-CNS cancers. We will then discuss the current trials of checkpoint inhibitors in GBM and the rationale for combining them with radiotherapy.

## The Immune System in the CNS

The immune system can be divided into two types of response, the innate (non-specific) and the adaptive (targeted) response. The latter relies on antigen presentation to T-cells by antigen-presenting cells (APCs) such as B-cells, macrophages, and dendritic cells (DC), while the former utilizes physical boundaries and non-specific inflammation. The CNS is protected by the blood–brain barrier (BBB), which consists of specialized endothelial cells joined together by tight junctions, as well as the glia limitans, a combination of astrocyte foot processes and basement membranes (6). The BBB serves to maintain CNS homeostasis and restrict entry of pathogens. The adaptive response in the CNS may be triggered in several ways: by the flow of soluble antigens to the draining deep cervical lymph nodes, where it is taken up by peripheral APCs; by DC near the meninges; or by microglia (the APC intrinsic to the CNS) (7). This differs from the peripheral immune system, where APCs migrate from the source of inflammation carrying the antigen to the lymph nodes or spleen, at which point antigen presentation occurs to T- and B-cells. Moreover, the neurons and astrocytes in the non-diseased state have an immunosuppressive action mediated by programmed cell death 1 (PD-1) and co-stimulatory molecule B7 homologs that restrict antigen presentation and downregulate T-cell function (8). However, in the diseased state, the BBB is disrupted, which allows immune cells greater access to the CNS.

**Figure 1:**
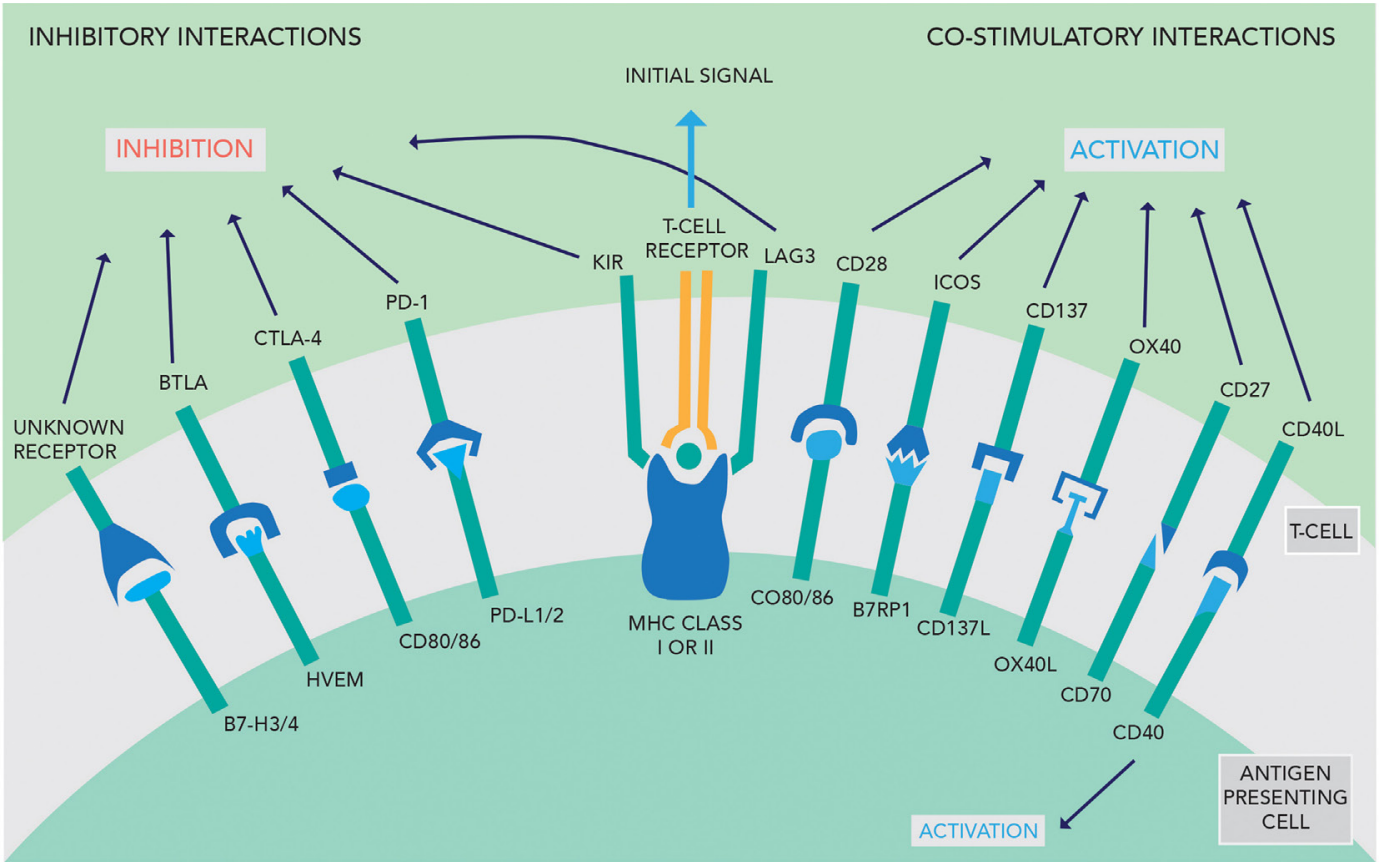
An overview of the inhibitory and co-stimulatory interactions between antigen-presenting cells and T-cells [adapted with permission from Macmillan Publishers Ltd., copyright 2012 (3)].

The activity of the immune system is tightly regulated by stimulatory and inhibitory interactions between the APC and T-cells. To activate T-cells, the APC must present the foreign antigen to the T-cell receptor. This interaction in isolation causes T-cell anergy. Further stimulatory signals from the APC are required to activate the T-cell, such as activation of CD28 by CD80/86 (see Figure 1). Conversely, if the APC expresses ligands that activate CTLA-4, this inhibits T-cell activation. The perpetuation of any T-cell response depends in part on the PD-1/PD-L1 interaction—if this occurs, then effector T-cells are unable to mount a persistent immune response (9). These competing signals act as checkpoints to prevent immune hyperstimulation or autoimmunity while generating a proportionate response to pathogens.

Cancer cells produce tumor antigen that can generate an immune response, as shown by the presence of tumor-infiltrating lymphocytes (TILs) into many solid tumors, including GBM (10, 11). However, T-cell activation is limited by both the recruitment of T-regs (expressing CTLA-4) into the tumor stroma and the suppression of any activated T-cells by GBM expression of PD-L1. In addition, tumor-infiltrating macrophages (which can constitute as much as 33% of the cell mass of a GBM) appear to have an immunosuppressive effect governed by the signal transducer and activator of transcription 3 (STAT3) pathway. Glioma cells express high STAT activity (12); therefore, tumor immunosuppression likely arises from effects on both T-cells and macrophages. The role of the CTLA-4 and PD-1 checkpoints has been investigated in GBM, as detailed below.

### Cytotoxic T-Lymphocyte-Associated Protein 4

Cytotoxic T-lymphocyte-associated protein 4 acts as a negative signaling antigen on activated T-cells, memory T-cells, and T-reg cells, competing with CD28 to interact with CD80 and CD86 on other T-cells (see Figure 1) (13). T-regs are CD4+ T-cells expressing the FoxP3 gene and appear to be the main coordinator of immunosuppression using CTLA-4 signaling (14, 15). Chambers et al. hypothesized that CTLA-4 acts *via* a threshold mechanism, lowering the immune response by altering the activation threshold for T-cell activation and decreasing clonal expansion (16).

Tivol et al. showed that mice deficient in CTLA-4 cannot negatively regulate T-cell proliferation leading to lymphoproliferative disorders and death (17). Fecci et al. have reported a correlation between an increased T-reg fraction and defects in CD4 cell proliferation in GBM. This study analyzed peripheral blood and tumor samples from GBM patients (*n* = 20) and healthy volunteers (*n* = 10). In GBM patients, the overall CD4+ T-cell numbers were decreased in both peripheral blood and the tumors compared to controls, but the fraction of T-regs within the CD4+ population was 2.63 times greater in the GBM group (18). Jacobs et al. demonstrated that GBM-infiltrating T-regs have high expression of CCR4, which is a receptor for the glioma-secreted chemokines CCL2 and CCL22, which may explain the increase in T-regs in glial tumors (19, 20). The constitutive expression of CTLA-4 on T-regs and their increase in GBM patients raises the possibility that anti-CTLA-4 monoclonal antibodies (e.g., ipilimumab) can be used for therapeutic benefit. However, a study of ipilimumab in melanoma and prostate cancer found that there were more FoxP3-positive (therefore immunosuppressive) T-regs in cancer patients treated with ipilimumab than in untreated patients without a cancer diagnosis suggesting that the mechanism of action of CTLA-4 is yet to be fully explained (21).

### Programmed Cell Death Ligand 1

Programmed cell death ligand 1 (see Figure 1) is the ligand of PD-1 and may be expressed on normal T-cells, B-cells, DC, and natural killer cells, as well as non-lymphoid tissue (14). An immunohistochemical study in GBM specimens found PD-L1 expression was prevalent with 60% of samples having at least 1% or more positive cells. In addition to staining on GBM cells, PD-L1 expression was found on lymphocyte-like cells, representing up to 28.6% of the positive cells counted. Moreover, GBM patients from the same study with high PD-1 and PD-L1 expression had worse survival outcomes, with an overall survival of 6.21 months shorter than those with low expression (22). In addition, Wintterle et al. found PD-L1 protein expression in both GBM (*n* = 9) and WHO Grade II mixed glioma (*n* = 1) specimens. The authors also found that PD-L1 is expressed constitutively at low levels in many malignant glioma cell lines (4). Moreover, Parsa et al. suggest that PD-L1 expression may be upregulated in certain glioma cell lines and a small number of GBM tissue specimens with a PTEN gene mutation or deletion, which is associated with a worse prognosis (23, 24).

## Immune Checkpoint Inhibitors in Non-CNS Cancers

### CTLA-4 Inhibitors

Ipilimumab, an anti-CTLA-4 monoclonal antibody, has shown efficacy in metastatic melanoma. A phase 3 study combining ipilimumab and the alkylating agent dacarbazine was compared to treatment with placebo and dacarbazine. Median overall survival increased from 9.1 months in the dacarbazine group to 11.2 months with combination therapy (25). However, in small cell lung cancer, the addition of ipilimumab to platinum and etoposide was of no additional benefit in terms of overall survival (26). This highlights how immunotherapy has variable effects across tumor types.

### Anti PD-1 Monoclonal Antibodies

Nivolumab, a PD-1 inhibitor, has also been extensively used as an immunotherapeutic agent in several cancers with good efficacy. In untreated melanoma patients without a BRAF V600E mutation, nivolumab treatment alone had a 72.9% overall survival at 1 year compared to 42.2% with dacarbazine treatment (27). Treatment with nivolumab has also been investigated in recurrent lung cancer. A 3-month survival benefit was observed in both squamous and non-squamous lung cancer when nivolumab was compared to docetaxel (28, 29). In advanced renal-cell carcinoma patients who had previously undergone antiangiogenic therapy, nivolumab was compared to everolimus, an mTOR inhibitor (2). This trial showed a median overall survival of 25.0 months in the nivolumab group (*n* = 406) compared to 19.6 months in the everolimus group (*n* = 397).

These trials have all looked at the possibility of using PD-1, or its ligand PD-L1, as a predictive marker of response. In melanoma, there was no correlation between response and PD-1 expression. This was also the case for squamous lung cancer and renal-cell cancer. However, in non-squamous lung cancer increased PD-L1 expression was indicative of a better outcome, indicating that in some cancer types determining PD-1 or PD-L1 expression may be beneficial to determining optimal treatment. The combination of nivolumab and ipilimumab compared against both agents alone has been assessed in a phase 3 trial in metastatic melanoma (30). Combination treatment gave a PFS of 11.5 months, nivolumab alone 6.9 months, and ipilimumab alone 2.9 months. The combination resulted in increased toxicity with 55% of patients experiencing grade 3 or 4 side effects. It does however highlight the additional efficacy of combining different checkpoint inhibitors in the treatment of cancer.

Newer PD-1 inhibitors have also become available. Pembrolizumab is a high-affinity humanized PD-1 monoclonal antibody, which in a phase 3 study was compared with ipilimumab treatment in melanoma patients with BRAF V600E mutations (31). Pembrolizumab had an overall response rate of 33.7% compared to 11.9% in the ipilimumab group and an increased median PFS of 5.5 months (*n* = 279) compared to 2.8 months in the ipilimumab group (*n* = 278).

### Anti-PD-L1 Monoclonal Antibodies

PD-1 binds to the checkpoint molecule PD-L1. In addition to inhibiting PD-1, an alternative strategy has been to develop antibodies against PD-L1. One such monoclonal antibody is atezolizumab, which has been investigated in metastatic renal-cell carcinoma. A phase 1a trial showed that the drug was well tolerated in a group of 70 patients (32). In non-small cell lung cancer, durvalumab, a human IgG1 monoclonal antibody against PD-L1, has been trialed in a phase 1b study in combination with tremelimumab, a monoclonal antibody targeting CTLA-4 (33). Clinical activity was noted regardless of PD-L1 status, and the combination of drugs was tolerated manageably. Phase 3 trials are currently ongoing in these new drugs and hopefully will provide additional treatment options.

### Current Immunotherapy Trials in GBM

The rationale for the use of checkpoint inhibitors in GBM is to harness the specificity of the adaptive immune response by blocking the tumor-induced inhibition of the T-cell response thereby promoting immune-mediated cytotoxicity. There is one large unblinded randomized phase 3 trial looking at nivolumab in newly diagnosed MGMT unmethylated GBM, CHECKMATE 498, which has just closed to recruitment. This compares the standard Stupp regimen of temozolomide with radiotherapy, with nivolumab and radiotherapy, with overall survival as the primary endpoint. Temozolomide has been omitted from the experimental arm due to the small benefit in unmethylated patients and potential negative effect of chemotherapy on the immune system. There is also a double-blinded sister study, CHECKMATE 548, in methylated MGMT patients, where both arms are treated with the Stupp regimen, and nivolumab is compared to placebo. This trial is currently recruiting and is looking at overall survival as the primary endpoint. There are three current smaller trials using another PD-1 inhibitor, pembrolizumab. The first is a phase 2 study in recurrent GBM (NCT02337491) combining pembrolizumab with VEGF inhibitor bevacizumab versus pembrolizumab alone (34). Preliminary results show safety and tolerability of pembrolizumab in combination with bevacizumab, with a median overall survival of 6.8 months in a cohort of six patients. Another phase 2 trial, also in recurrent GBM (NCT02337686), is giving all study participants the drug before surgical resection and then continual doses after surgery until disease progression in order to obtain data on 6-month PFS and effector T-cell to T-reg ratio (35). In the third, pembrolizumab is also being trialed in newly diagnosed GBM, in a phase 1/2 study (NCT02530502) looking at pembrolizumab in combination with standard temozolomide and radiation treatment to ascertain dose-limiting toxicity and 6-month PFS (36).

Programmed cell death ligand 1 inhibitors are also being currently investigated in early clinical trials. There are two current open phase 1 and phase 2 studies (NCT01375842 and NCT02458638) looking at atezolizumab in solid cancers including GBM, the results of which are awaited (37, 38). An ongoing phase 2 study (NCT02336165) in GBM is looking at durvalumab in GBM both as monotherapy and in combination with bevacizumab, with primary endpoints of clinical efficacy, overall survival, and 6-month progression-free survival (39).

### Combination Immunotherapy in GBM

A review by Intlekofer and Thompson has put forward a rationale for combined blockade of CTLA-4 and PD-1 by suggesting that this could increase the immune response to the tumor by lowering the threshold of T-cell activation and enabling clonal expansion while simultaneously increasing effector T-cell function (14). Indoleamine 2,3-dioxygenase (IDO) is one of several immune checkpoints involved in tumor immune escape. The IDO enzyme, activated in DC and macrophages, helps create an environment that favors suppression and tolerance. Wainwright et al. showed therapeutic inhibition of IDO, CTLA-4, and PD-L1 in a mouse model of well-established glioma maximally decreases tumor-infiltrating T-regs, coincident with a significant increase in T-cell-mediated long-term survival (40). In fact, 100% of mice bearing intracranial tumors were long-term survivors following triple combination therapy.

Bristol-Myers Squibb is looking at the efficacy and safety of nivolumab versus bevacizumab and nivolumab with or without ipilimumab in GBM in a recruiting phase 3 study (CHECKMATE 143). This trial assesses the combination of two modes of immunotherapy while also looking to compare the efficacy of immunotherapy with antiangiogenic therapy. The primary endpoints are safety and tolerability in cohorts receiving either nivolumab alone or nivolumab and ipilimumab, and overall survival in the arm comparing nivolumab treatment to bevacizumab treatment. Secondary endpoints are overall survival at 12 months, progression-free survival, and objective response rate. Preliminary results as expected show that treatment with nivolumab alone is better tolerated than combination treatment with nivolumab and ipilimumab (41). A phase 2 study (NCT02794883), looking at combination therapy with durvalumab and tremelimumab in recurrent malignant glioma, will provide an interesting parallel to CHECKMATE 143 by targeting PD-L1 and CTLA-4 instead of PD-1 and CTLA-4 (42).

### The Potential for Immunotherapy and Radiotherapy in Glioma

The presentation of tumor antigen by APCs to T-cells provides the initial signal for the immune system to target malignant cells, and CTLA-4 or PD-1/PD-L1 antibodies serve to disinhibit the response. A complementary strategy would be to enhance the immunogenicity of the tumor. Ionizing radiation achieves this in multiple ways: release of pro-inflammatory cytokines (such as interferon-γ), increased production and variety of tumor-associated antigens, and expression of molecules on tumor cells that make them susceptible to T-cell-mediated killing (calreticulin, MHC class 1, CD95, and NKGD2) (15, 43). Fractionated radiotherapy can induce PD-L1 expression in tumor cells and enhance immune escape, which could be modified by an anti-PD-L1 antibody (44). In addition, radiation increases the ratio of T-effector cells compared to T-reg cells and promotes infiltration of T-cells into the tumor microenvironment, not only by inducing inflammation but also by direct vascular damage that allows DC to access tumor antigen and to mature into APCs (45, 46). Radio-immunotherapy potentially could tip the balance in the tumor environment from immunosuppression to immune activation.

The abscopal effect, the uncommon phenomenon seen clinically where tumor shrinkage occurs at a location distant from the area irradiated, is likely to be a result of the above processes (47). Mouse models suggest that this effect is mediated by T-cells and DC (48). Adding a CTLA-4 inhibitor enhanced the abscopal effect in mice inoculated with breast cancer cells in two sites, where only one site was irradiated (49). In glioma cells injected into mouse striatum, the combination of CTLA-4 blockade, 4-1BB activation (4-1BB, when activated, stimulates CD8+ T-cell proliferation), and radiotherapy significantly improved overall survival compared to a single modality alone (50). Subsequent biopsies confirmed a significantly higher number of CD4+ and CD8+ T-cells in the tumors that received all three treatments. Similarly, in mice implanted with glioma cells, radiotherapy with an anti-PD-1 antibody resulted in a long-term cure rate of 15–40%, while neither treatment alone produced long-term responses (45). As GBM cells can migrate throughout the CNS and therefore outside of a conventional radical radiotherapy field, using immunotherapy to potentiate the abscopal effect is an attractive possibility (51).

Human trials testing this concept are ongoing. A phase 3 trial, in the setting of metastatic prostate cancer, compared the use of ipilimumab alone against ipilimumab and radiotherapy and failed to demonstrate an overall survival benefit (52). Phase 1 and 2 studies in multiple different tumor sites continue, mainly with CTLA-4 blockade (or other novel immune targets such as OX40) with radiotherapy (53). Combination therapy in the CNS has several theoretical risks such as immune hyperstimulation in a tissue that can ill tolerate excess inflammation and damage. The usual milieu of the CNS is immunosuppressive, and to stimulate the immune system to overcome this may increase the risk of autoimmune damage elsewhere in the body. In addition, while checkpoint inhibitors may increase the activity of TILs, it is unclear how much this will affect tumor-associated macrophages and microglia. It is unclear what the ideal dose and fractionation of radiotherapy is required to generate an immune effect *in vivo*. The overall effect in humans of multimodality immune manipulation is difficult to predict, and we await the results of the ongoing research.

## Conclusion

**Figure 2:**
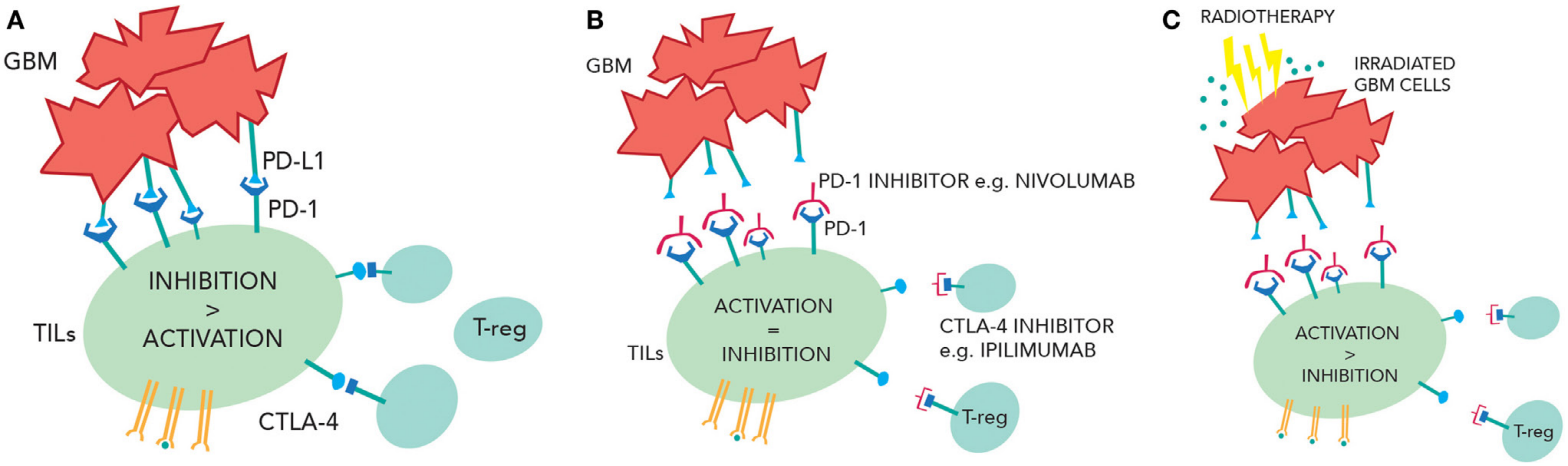
**(A)** Inhibition of the tumor-infiltrating lymphocytes (TILs) due to PD-1/programmed cell death ligand 1(PD-L1) interactions and cytotoxic T-lymphocyte-associated protein 4 (CTLA-4) interactions. **(B)** Checkpoint inhibition of CTLA-4 and PD-1 reduce TIL suppression and increase TIL activity. **(C)** Radiotherapy releases more tumor antigens causing greater TIL activation. In the context of checkpoint inhibition, this may cause more immune-mediated cell death.

Immunotherapy is now an established modality of treatment for melanoma and lung cancer and is under investigation in CNS tumors. GBM cells express inhibitory signals that prevent the immune system from mounting a significant antitumor response, and ongoing clinical trials will assess whether PD-1 and/or CTLA-4 inhibitors can overcome this. Radiotherapy potentially could increase numbers of active TILs in the CNS (by enhancing antigen presentation, increasing tumor immunogenicity, and making the BBB more porous) while immunotherapy could block the inhibition of TILs by GBM cells and T-regs, creating a larger response in the tumor (Figure 2). This approach may not be effective because of access of drugs or T-cells into the CNS, the prevailing immunosuppressive environment of the brain, or anatomical differences of the immune system in the CNS. Moreover, the combination of treatment could provide immune hyperstimulation and unacceptable toxicity. However, given the unacceptable prognosis of GBM, this novel approach is worthy of further research.

## Author Contributions

NL and RR were responsible for writing the text and reviewing the research papers. RR is responsible for the figures. SN and KK are responsible for the conception of this mini-review, editing the text, and reviewing the research papers.

## Conflict of Interest Statement

The authors declare that the research was conducted in the absence of any commercial or financial relationships that could be construed as a potential conflict of interest.
